# Eliciting Specific Electrochemical Reaction Behavior by Rational Design of a Red Phosphorus Electrode for Sodium-Ion Batteries

**DOI:** 10.3390/nano11113053

**Published:** 2021-11-13

**Authors:** Jong Hyuk Yun, San Moon, Do Kyung Kim, Joo-Hyung Kim

**Affiliations:** 1 Department of Materials Science and Engineering, Korea Advanced Institute of Science and Technology, Daejeon 34141, Korea; jh.yun@kaist.ac.kr; 2 Advanced Materials Division, Korea Research Institute of Chemical Technology, Daejeon 34114, Korea; san82@krict.re.kr; 3 School of Materials Science and Engineering, Gyeongsang National University, Jinju 52828, Korea

**Keywords:** red phosphorus, sodium-ion battery, alloying reaction, reaction mechanism

## Abstract

Due to the demand to upgrade from lithium-ion batteries (LIB), sodium-ion batteries (SIB) have been paid considerable attention for their high-energy, cost-effective, and sustainable battery system. Red phosphorus is one of the most promising anode candidates for SIBs, with a high theoretical specific capacity of 2596 mAh g^−1^ and in the discharge potential range of 0.01–0.8 V; however, it suffers from a low electrical conductivity, a substantial expansion of volume (~300%), and sluggish electron/ion kinetics. Herein, we have designed a well-defined electrode, which consists of red phosphorus, nanowire arrays encapsulated in the vertically aligned carbon nanotubes (P@C NWs), which were fabricated via a two-step, anodized-aluminum oxide template. The designed anode achieved a high specific capacity of 2250 mAh g^−1^ (87% of the theoretical capacity), and a stepwise analysis of the reaction behavior between sodium and red phosphorus was demonstrated, both of which have not been navigated in previous studies. We believe that our rational design of the red phosphorus electrode elicited the specific reaction mechanism revealed by the charge–discharge profiles, rendered excellent electrical conductivity, and accommodated volume expansion through the effective nano-architecture, thereby suggesting an efficient structure for the phosphorus anode to advance in the future.

## 1. Introduction

Among the great efforts that are underway to improve the global future of energy, renewable energy is the most sustainable solution for many social and environmental problems [1]. Recently, with rapid developments in the electrical, electronic, and information communication fields, the demand for small portable devices such as smartphones and notebooks is dramatically increasing. In addition, the demand for energy storage systems and electric vehicles is emerging in response to environmental and energy issues. Rechargeable secondary batteries that can be continually charged and discharged using renewable energy sources instead of fossil fuels are representative of the eco-friendly, green technologies that could greatly reduce the dependence on fossil fuels and reduce carbon dioxide emissions. At the same time, the demand for improved performance is also significantly increasing. In recent decades, as demand for high-energy rechargeable batteries has steadily grown, advanced sodium-ion batteries (SIBs) have been intensively studied as an attractive option for storing electrical energy, due to the natural abundance of sodium, and its price advantages over lithium. In particular, there has been considerable interest in large energy storage systems (ESS) due to the need to go beyond the limits of lithium-ion batteries (LIBs) [2,3,4,5].

SIBs need high-level physicochemical properties (high energy density, long-term durability, high Coulombic efficiency, minimal charging time, low cost, high mechanical/chemical reliability, eco-friendliness, etc.) to satisfy the demanding performance requirements of various fields [6,7]. Graphite and its intercalation-type analogs have the greatest renown as excellent anode materials in LIBs; however, it was found that sodium ion is difficult to intercalate between the graphene layers of the electrode because of its 25% larger radius than lithium ion [8,9]. Other intercalation-type anodes, including TiO_2_, NaTi_2_(PO_4_)_3_, and Na_4_Ti_5_O_12_, can be regarded as good alternatives, but the limited number of Na^+^ ions involved in the electrochemical reaction has impeded their practical use [10,11,12]. Inspired by these points, extensive research on the electrochemistry of various transition metals (e.g., Sn, Sb, Ge, Si) has been performed to find anode materials that provide high energy density [13,14]. Since a large number of Na^+^ ions can alloy with the transition metal and the working potential is low, the alloying-type anodes for SIBs have succeeded in promoting the specific capacity, despite that, the large volume expansion resulting in severe mechanical strain has not been surmounted [15]. In this regard, another category of “conversion-type” anode materials has been on the rise due to the materials’ excellent specific capacity and the wide range of material options [16].

Phosphorus is one of the most promising conversion-type anode candidates [17,18], and provides a high theoretical specific capacity (2596 mAh g^−1^), corresponding to final phase Na_3_P. Furthermore, the operating voltages in the range of 0.0–0.8 V have been demonstrated using phosphorus-based materials, which guarantees a high energy density, although the redox potential of Na/Na^+^ is −2.71 V versus a standard hydrogen electrode (+0.3 V of lithium) [19,20].

As a promising negative-electrode candidate offering high energy density, useful optimization studies of phosphorus’s electrochemical performance have been performed. However, in-depth investigations to establish a fundamental understanding of phosphorus in this role have not been performed. Thus, despite its promise as an anode material for SIBs, there are still several challenges to the use of phosphorus, such as a low electrical conductivity (ρ < 1 × 10^−14^ S cm^−1^), a fatal capacity-fading due to volume expansion, and its largely irreversible capacity after being fully sodiated to the Na_3_P phase during the alloying reaction process [21,22]. Furthermore, phosphorus has three allotropes (white, red, and black phosphorus). White phosphorus begins to ignite in air at 30 °C, so it is difficult to analyze at an atomic scale the fundamental reaction mechanism of the phosphorus electrode, due to surface oxidation. In addition, it is not suitable in terms of electrode fabrication, and has poor safety in air. In contrast, red phosphorus has good chemical stability at room temperature and atmospheric pressure, and its physicochemical properties include an acceptable sodium ion conductivity and a high electrochemical performance [23].

To improve the fundamental properties of phosphorus, it has been combined with carbon and utilized as an anode material for SIBs. Red phosphorus-carbon nanotube (CNT) hybrid nanocomposites with a reversible capacity of 1675 mAh g^−1^, a capacity retention of 76.7% over 10 cycles, and with facile processing at a low cost, have been synthesized by physical mixing and subsequent annealing [24]. This suggests the potential for utilizing red phosphorus-CNTs. However, because the phosphorus is inhomogeneously distributed on the CNT surface, irreversibility is exhibited and sufficient electrical conductivity is not assured, and an electrical potential drop (i.e. iR drop) has been clearly observed in the initial reaction region.

To avoid the ignition of red phosphorus, Li et al. used delicate thermal processing to synthesize a red P@CMK-3 hybrid material by melting-diffusion under inert gas. Sequential thermal treatment by infiltrating phosphorus into CNTs at 450 °C, with a reversible conversion at 260 °C, was implemented in a sealed vessel [25]. The hybrid composite facilitated volume expansion of the phosphorus during sodiation/de-sodiation and also offered a high electron conductivity. However, the reversible specific capacity of red phosphorus was 1020 mAh g^−1^, only about 40% of its theoretical capacity. It can be inferred that the irreversible reaction could be eliminated by a conversion process at low temperature, but the usable amount of red phosphorus would decrease at the same time. Those findings led researchers to nanosize red phosphorus and confine it to the conductive matrices [26,27,28,29]. The recent efforts have reinforced the specific capacity (>1000 mAh g^−1^) and the cycling ability of the red phosphorus anodes, yet achieving the theoretical capacity remains as the challenge. The nano-architecture of the red phosphorus anodes needs to be advanced to conceive the electrochemistry between sodium and red phosphorus necessary to accomplish the high-performance anode. Therefore, to study the fundamental electrochemical behavior of red phosphorus, it is necessary to provide 3D carbon nanostructures that completely enclose the electrode material [30,31]. In the work reported in this paper, we fabricated 3D-aligned, red-phosphorus nanowires with carbon nanowalls using a combinational two-step anodization and a chemical vapor deposition process. Using these processes, we anticipate that the resulting phosphorus electrodes will have many synergistic structural advantages for advanced 3D SIB architectures. A high specific capacity of 2250 mAh g^−1^ (87% of the theoretical capacity) was achieved, and a stepwise analysis of the reaction behavior via charge-discharge profiles was demonstrated, both of which have not been navigated in previous studies. With an in-depth understanding of the electrochemical reactions of red phosphorus, we aim to present guidelines for determining the effect of these 3D nanostructures for the analysis of future SIB-anode materials.

## 2. Materials and Methods

Preparation of Porous Template: The porous alumina templates were prepared using a two-step anodization of aluminum discs in 0.3 M oxalic acid at 25 °C. High-purity aluminum foil (99.999%, Sigma Aldrich, St. Louis, MO, USA) was used as the working substrate. First, the foil was cleaned in an ultrasonic bath of acetone and then annealed at 400 °C for 3 h in nitrogen (N_2_). Next, the sample was electropolished in a 4:1 (*v/v*) mixture of ethanol and perchloric acid (HClO_4_, Sigma Aldrich, St. Louis, MO, USA) subjected to 20 V for 2 min to minimize the roughness of the aluminum foil surfaces. A two-step anodization was then performed to create self-ordered porous alumina on the aluminum surface. The first anodization was carried out in 0.3 M oxalic acid at 40 V and 20 °C for 5 h. The alumina template was then removed by wet chemical etching in a mixture of 0.4 M phosphoric acid (H_3_PO_4_, Sigma Aldrich, St. Louis, MO, USA) and 0.2 M chromic acid (H_2_CrO_4_, Sigma Aldrich, St. Louis, MO, USA) at 60 °C for 2 h. The second anodization was based on the same conditions, but the anodizing time was 20 min. Finally, the alumina templates were etched further to widen the pore diameters by dipping them in 6 wt% phosphoric acid for 60 min.

Preparation of Aligned Red P@C NWs: Carbon nanotubes (CNTs) were prepared by depositing carbon layers on the pore surfaces of the alumina template using CVD. The alumina templates were placed in a tubular furnace with their pores aligned along the horizontal tube direction. Next, the furnace was heated to 640 °C in a flow of nitrogen (100 sccm). At this temperature, a flow mixture of C_2_H_2_ and N_2_ (10% C_2_H_2_ and 90% N_2_) was maintained for 1.5 h and the furnace was then cooled to room temperature under a N_2_ flow. For the infiltration of phosphorus (99.99%, Sigma Aldrich, St. Louis, MO, USA) into the membrane pores, the membrane was first placed in a sealed vessel and kept in red phosphorus at 450 °C for 3 h at a rate of 3 °C min^−1^ under Ar gas. After cooling to 300 °C at a rate of −1 °C min^−1^, the infilled membrane was kept at 300 °C for 18 h to convert the white phosphorus to red phosphorus. The excess phosphorus on the upper side of the membrane was removed. Next, the heat-treated alumina templates were attached to 316 stainless steel substrates using conductive epoxy (CW2400, Chemtronics, Kennesaw, GA, USA). The residual aluminum and alumina were removed with saturated HgCl_2_ and 3 M NaOH solution, respectively. The weight portions of the carbon and phosphorus were determined using an elemental analyzer (Flash 2000, Thermo Scientific, Waltham, MA, USA).

Preparation of Red P@CNT NCs: Red phosphorus and CNT were physically mixed to a mass ratio of 2:1. The mixture was placed in a sealed vessel. The infiltration process of red P@CNT nanocomposites followed the protocol used for the aligned red P@C nanowires.

Material Characterization: The X-ray diffraction pattern of the red phosphorus was confirmed by X-ray diffraction (XRD, D/MAX-RB 12KW, RIGAKU, Tokyo, Japan) with a wavelength of λ = 0.15418 nm over an angular range of 10° ≤ 2*θ* ≤ 70° at a step width of 0.01°. The microstructure and morphology of the red P@C nanowires were analyzed using scanning electron microscopy (SEM, XL30, Philips, Amsterdam, Netherlands) at an acceleration voltage of 10 kV and with transmission electron microscopy (TEM, Tecnai G2 F30 S-Twin, FEI, Hillsboro, OR, USA) operated at 300 kV. The vaporization-deposition temperature of red phosphorus was determined by differential scanning calorimetry (DSC, DSC 404 F1, NETZSCH, Selb, Germany), which was conducted from 25 to 550 °C at the heating rate of 10 °C min^−1^ in Ar atmosphere.

Electrochemical measurements: Electrochemical tests were carried out using a 2032 coin-type half-cell with Na metal as both the counter and reference electrodes. The batteries were assembled in an Ar gas filled glove box with H_2_O content < 0.3 ppm and O_2_ content < 0.1 ppm. The electrolyte was prepared by dissolving 1 M NaClO_4_ (98%, Sigma Aldrich, St. Louis, MO, USA) in propylene carbonate (PC) / fluoroethylene carbonate (FEC) (98:2 wt%) (Panaxetec, Busan, Korea). The glass fiber membranes (GF/D, Whatman, Maidstone, UK) were used as separators. Cyclic voltammetry was performed using a multi-channel battery tester (BioLogic VMP3, Seyssinet-Pariset, France) with a cut-off voltage range from 0.01 to 2.5 V (vs. Na/Na^+^) at a sweep rate of 0.05 mV s^−1^. The galvanostatic measurements were carried out within the range 0.01–2.5 V of potential (vs. Na/Na^+^) using a battery cycler (WBCS3000, WonATech, Seoul, Korea).

## 3. Results

### 3.1. Fabrication of Electrodes

**Figure 1:**
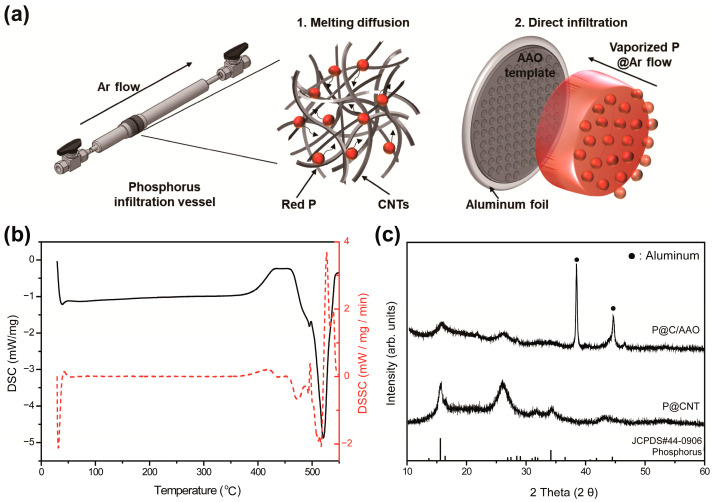
(**a**) Schematic illustration of methods to infiltrate red phosphorus into a carbon-based nanostructure; 1. Melting diffusion and 2. Direct infiltration, (**b**) differential scanning calorimetry (DSC) measurements of red phosphorus for determination of vaporization deposition temperature and (**c**) X-ray powder diffraction patterns (10–60°) with peaks in the aligned red P@C NWs (after infiltration of red phosphorus).

The red phosphorus@carbon nanocomposites were fabricated by two different methods to verify the relation between electrochemical properties and structural features, as shown in Figure 1a. The red phosphorus@CNTs nanocomposite was synthesized via simple mixing and a melting–diffusion process. In contrast, an electrode with a special ordered structure was fabricated by direct infiltration using a combination of phosphorus sublimation and argon flux. To investigate the vaporization temperature of the red phosphorus, differential scanning calorimetry (DSC) measurements were executed from 25 to 550 °C. Commercial red phosphorus exists in an amorphous phase, and it emits heat energy (from 410 to 450 °C) that promotes crystallization, as shown in Figure 1b. Therefore, the temperature of the melting–diffusion reaction was fixed at 450 °C when vaporization began. Both electrodes were fabricated using the same thermal protocol.

The X-ray diffraction patterns of the red phosphorus peak were identical, as shown in Figure 1c, and the sharp diffraction peaks between 22° and 28° suggest that CNTs retained sufficient crystallinity after the thermal process. Additionally, the peaks were indexed as red P@C NWs: detected at 2*θ* = 13°, 15°, 16°, 27°, 28°, 29°, and 31°, corresponding to (−111), (013), (004), (212), (11−7), (030), and (−218); and a P2/c monoclinic space group (Joint Committee on Powder Diffraction Standards, JCPDS No. 44-0969).

**Figure 2:**
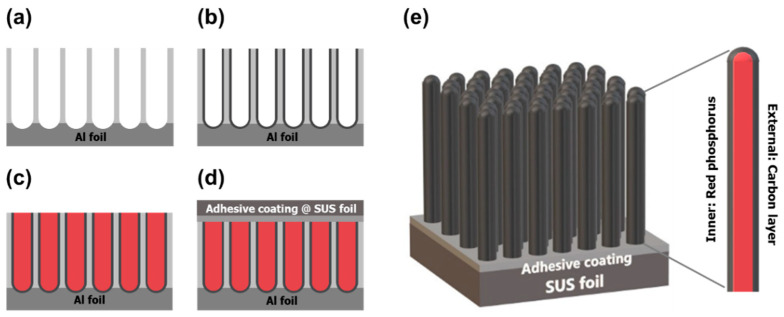
Schematic illustration of the process for fabrication of the aligned red phosphorus@carbon nanowires: (**a**) preparation of porous alumina templates by a two-step anodization process, (**b**) carbon layer deposition by a CVD process to form an array of CNTs, (**c**) phosphorus infiltration utilizing a melting-diffusion process through heat-treatment at 450 °C, (**d**) adhesive coating on the opposite side and attachment with SUS foil, and (**e**) final aligned red P@C nanowires after removal of both the aluminum foil and alumina by a wet-etching step.

Aligned red phosphorus@carbon nanowires (red P@C NWs) were successfully synthesized using a two-step anodized-aluminum oxide (AAO) template and a subsequent chemical vaporization deposition process to construct a carbon shell. The anodization process is the most critical step for defining the aspect ratio of the phosphorus core of the final red P@C electrode [32,33]. In our case, we engaged a two-step anodization process to generate aligned pore channels (as shown in Figure 2a) while the channel lengths were kept at ~3 μm. We also used an additional pore-widening step to increase the pore diameter to facilitate a higher efficient phosphorus infiltration than that of previous cases based on nano or mesoporous carbon, as well as to increase the sulfur content as much as possible. In the same context of high specific capacity, by tuning the CVD duration, the carbon thickness was kept to a minimal value of ~3 nm, as shown in Figure 2b. Figure 2c shows the facile melting–diffusion procedure including the infiltration of red phosphorus into the hollow carbon nanotubes, and the reductive annealing step to process the residual white P into red P. After the addition of an adhesive coating between the stainless-use steel (SUS) foil and the upper side of the template, a subsequent solution etching was executed, as shown in Figure 2d. SUS foil and the conductive adhesive maintain the carbon nanotube arrays while the residual alumina and aluminum etch out, and then serve as a current-collecting substrate enabled thorough encapsulation. The final, aligned red P@C NWs are illustrated in Figure 2e.

Figure S1 shows the two-step anodizing process used to prepare the red P@C nanowires. To remove residual particles on the aluminum surface, an electropolishing process was first executed, followed by the first anodization step (Figure S1a). A homogeneous pinch was formed by applying a constant voltage to the aluminum foil for a sufficient time (Figure S1b). After etching the alumina membrane using a basic solution, concave holes with extreme similarity were observed (Figure S1c). Finally, after the second anodizing process, these provided a remarkably uniform AAO template, as shown in Figure S1d.

**Figure 3:**
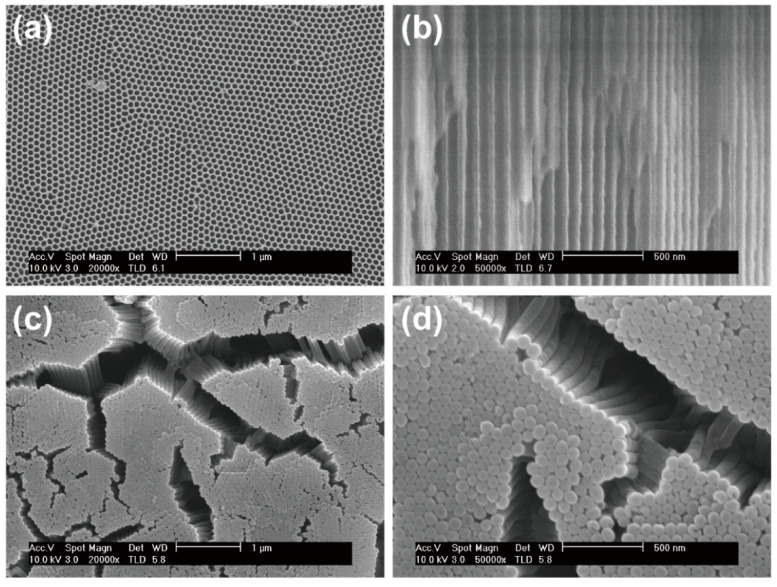
SEM images of the red P@C NWs electrode: (**a**) top-view and (**b**) cross-sectional view after the pore-widening process and carbon layer deposition by a CVD process to form an array of CNTs. Pt deposition on the opposite side and heat treatment at 400 °C. The final electrode structure after removal of the membrane by a wet etching step. A top-view SEM image of the red P@C NWs at (**c**) low and (**d**) high magnification.

The SEM images in Figure 3a show the topside views of the fabricated, carbon-coated AAO templates. These were composed of a vertically aligned tubular structure with an average diameter of 75 nm, which provided a porous and highly conductive network for accommodating the red phosphorus. The cross-sectional SEM image in Figure 3b clearly confirms that all nanowires are vertically aligned, tubular, and well-defined parts of a porous template membrane. The SEM images in Figure 3c,d show top and side views of the highly dense, red P@C nanowires, which constitute a forest-like structure (at low and high magnification, respectively). This indicates that the unique nano structure has uniform channels, facilitating the presence of red phosphorus within the conductive carbon wall, and that this nanostructure was retained, even after being subjected to the various etching processes.

**Figure 4:**
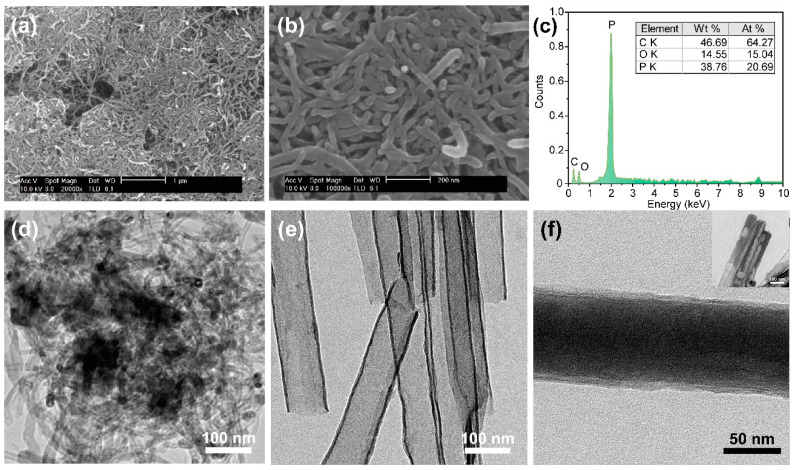
The microstructures of the red P@CNTs nanocomposites with (**a**) low and (**b**) high magnification, (**c**) elemental distributions, and (**d**) TEM image. The aligned CNTs (**e**) before and (**f**) after the infiltration of red phosphorus by the direct infiltration process.

Figure 4 shows the physical distribution of red phosphorus on the carbon matrix. The microstructure of the red P@CNT nanocomposites can be seen, with random distribution at low (Figure 4a) and high (Figure 4b) magnification. This indicates that the CNT surfaces were partially covered by red phosphorus and that the weight ratio of the electrode material is red phosphorus 38.76% to carbon 46.69%. This shows the difference from the initial experimental weight ratio (2:1), indicating there was a considerable loss of red phosphorus during the thermal process. Furthermore, it is expected that the condensed surface will present a serious obstacle to electrical conductivity, as shown in Figure 4d. To confirm the infiltration of red phosphorus into the tubular structures, we observed the microstructure of the hollow carbon nanotubes before and after the direct infiltration process. In Figure 4e, the thickness of the carbon-shell layer is about 3 nm, thus verifying the well-controlled CVD process used for carbon deposition. After the infiltration process, a part of the nanotubes was successfully filled with red phosphorus in close contact with the carbon layer (see Figure 4f). However, nanowires with incomplete infiltration occurred intermittently (inset of Figure 4f) because the gas-phase phosphorus was not sufficiently transferred to the bottom of the CNTs due to their elongated structure. Although the total efficiency of the special process used to infiltrate phosphorus into the carbon nanotubes was about 30%, it is expected that the fundamental electrical properties of the as-infilled red phosphorus could be adequately overcome by structural distinction.

### 3.2. Electrochemical Characterization

**Figure 5:**
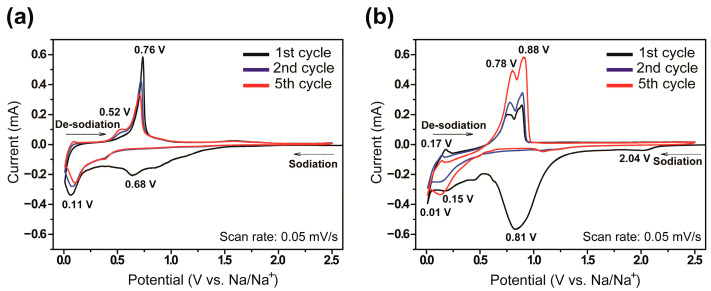
Cyclic voltammetry for the first (black), second (blue), and fifth (red) cycles of the red P@C electrodes at the scan rate of 0.05 mV s^−1^, ranging from 0.01–2.50 V. (**a**) Red P@CNT NCs and (**b**) aligned red P@C NWs.

The cyclic voltammetry (CV) of both phosphorus electrodes was first evaluated to investigate how the structural difference affected the electrochemical reactions for alloying sodium and phosphorus. The first, second, and the fifth CV profiles of the electrodes recorded in the range of electrical potential 0.01–2.5 V and at the scan rate of 0.05 mV s^−1^, which are shown in Figure 5a,b, respectively. The cathodic peak located at 0.81 V in the first cycle originated from the formation of a solid electrolyte interphase (SEI) layer caused by irreversible reactions (such as decomposition of the organic electrolyte) [26,28]. In the second sweep of the potential of the red P@C nanocomposites, two cathodic peaks were observed at 0.11 and 0.01 V, and two anodic peaks were observed at 0.52 and 0.76 V, as shown in Figure 5a [34,35].

In comparison, the anodic peaks of the aligned red P@C NWs, and, in particular, the two anodic peaks located at ~0.75–1.00 V in Figure 5b, can be clearly distinguished. Furthermore, two cathodic peaks located at 0.15 and 0.01 V, and three anodic peaks located at 0.17, 0.78, and 0.88 V all increased with additional cycling, indicating that the red phosphorus participates in the reversible reaction with sodium ions because of its structural homogeneity.

**Figure 6:**
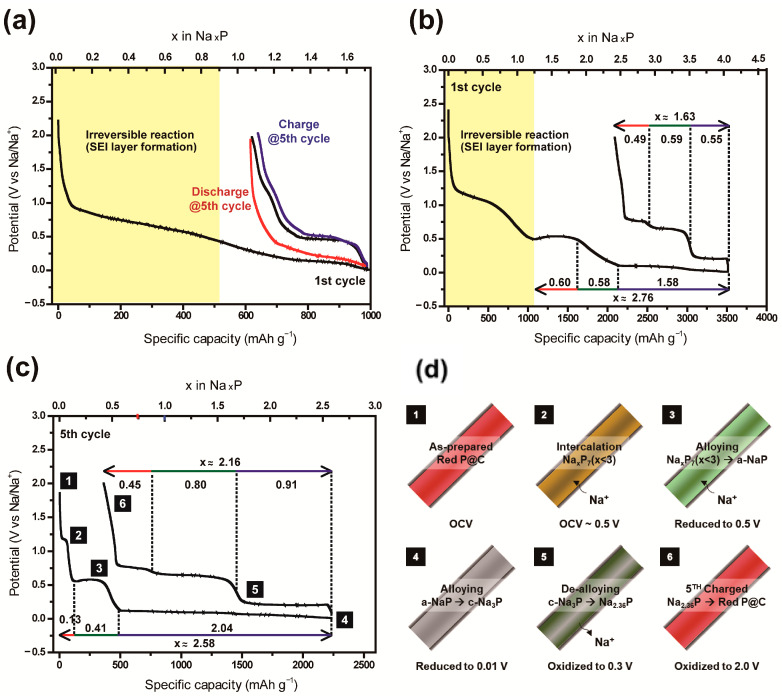
Galvanostatic charge/discharge voltage profiles of (**a**) red P@CNTs NCs between 0.01 and 2.00 V at a rate of 25 mA g^−1^, red P@C NWs between 0.01 and 2.00 V at a rate of 25 mA g^−1^ for the (**b**) first cycle and (**c**) fifth cycle. (**d**) The schematic illustration for electrochemical reaction of red P@C NWs.

The red P@C NCs showed a low specific capacity of about 1000 mAh g^−1^, due in part to side reactions such as electrolyte decomposition and SEI formation in the FEC added electrolyte, as shown in Figure 6a. The insignificant difference in the charge capacity of the first and fifth cycle indicates that a part of the red phosphorus participates in the reversible electrochemical reaction, but that most of the red phosphorus is not sodiated due to its biased atomic distribution. The aligned red P@C NWs exhibited (see Figure 6b) a high specific capacity of 3514 mAh g^−1^, but had poor cycle stability and a Coulombic efficiency of 83% due to the incomplete infiltration of phosphorus, as shown in Figure 6b. The first electrochemical reduction included the sodiation of the red P and about 30% irreversible reactions. The specific initial capacity of red P@C (loading of 0.50 mg of red P) reached 3513/1423 mAh g^−1^ in the first discharge/charge, respectively. Two plateaus appeared at potentials of 0.5 and 0.01 V, corresponding to the alloying with 0.60 and 1.58 sodium ions, respectively. These plateaus indicate a two-phase sodiation process into the phosphorus [36,37].

In addition, the curve with the range 1600–2100 mAh g^−1^ represents a single-phase reaction and exhibits a phase transformation according to the sodiation. The over-potential can be observed around 0.5 V, reflecting the alloying reaction of phosphorus with Na. The plateau of 0.5 V, related to the formation of NaP, shortened during cycling. However, the alloying reaction from NaP to Na_3_P at < 0.1 V was reversible. Remarkably, Figure 6c shows that the electrode demonstrates a reversible capacity of 2250 mAh g^−1^ in the fifth cycle. This is very close to the theoretical specific capacity of red phosphorus. The sodiation reaction of the aligned red P@C NWs had similar trends to that of the first cycle, even showing a more extended plateau region (both more reversible and participating in the electrochemical reaction). In the de-sodiation reaction of the fifth cycle, the expected phase of the Na-P alloy was close to that of Na_2.36_P because of the plateau at 0.3 V, which corresponds to alloying with nearly 0.91 sodium ions [38]. In contrast, the red P@C NCs showed capacity fading in subsequent cycles because of an imperfect coverage of phosphorus on the CNT surfaces. There are several reasons why the red P@CNTs NCs exhibited poor electrochemical performance: (i) detachment of the phosphorus from the CNTs (due to volume expansion), (ii) increase in side reactions (irreversible SEI layer formation and ignition of the phosphorus in air), and (iii) Na electrodeposition at a voltage near 0.01 V. The clearly distinct sodiation/de-sodiation process of the red P@NWs was attributed to their special structural advantages, such as the uniform distribution of phosphorus, diffusion control of Na ions, electronic conductivity secured in thin carbon layer, etc.

Galvanostatic curves, which are directly related to various intermediate states (NaP_7_, Na_3_P_7_, NaP, Na_5_P_4_, and Na_3_P), were not experimentally realized in a previous report [37,39,40]. Fundamental research of this kind could be achieved due to the remarkable nanostructures proposed herein, which are capable of overcoming volume expansion, improving the electron paths, and improving the sluggish sodium-ion kinetics [41,42,43]. Using the special structures of other electrode materials, it is possible to confirm unexpected intermediate phases and to measure their properties. In addition, advanced in situ or ex situ characterization studies could reveal that changes in the oxidation state of the sodiated phosphorus phases during the discharge/charge process offer a special strategy, and provide efficient storage of phosphorus in sodium.

## 4. Conclusions

Aligned red phosphorus@carbon nanowires were successfully synthesized using a two-step, anodic-anodized oxide template and a vapor-deposition process. These were used to evaluate red P@C NWs as a promising anode material for high-performance SIBs. This is the first experimental realization of the intermediate reaction during sodium insertion/de-insertion into the red P@C NWs using galvanostatic discharge/charge curves. The aligned red P@C NWs exhibited a high specific capacity of 2250 mAh g^−1^ with a Coulombic efficiency of 83%. The in-depth investigation of the aligned red P@C NWs revealed a distinctive reaction region of the intermediate states (NaP_7_, Na_3_P_7_, NaP, Na_5_P_4_, and Na_3_P) not reported in previous reports. Overall, our results present a promising strategy for utilizing red phosphorus as an anode material for SIBs. They also contribute to the understanding of the sequential intercalation-alloying reactions of red P@C NWs for use in high-performance sodium-ion batteries.

## Data Availability

Not applicable.

## Supplementary Materials

The following are available online at https://www.mdpi.com/2079-4991/11/11/3053/s1, Figure S1: Procedure for fabrication of the red P@C NW electrode: (a) electropolishing of the aluminum surface, (b) preparation of porous alumina templates by the first-step anodization process, (c) alumina etching process, and (d) preparation of porous alumina templates by a second-step anodization process.
