# Supplementary material for: Eliciting Specific Electrochemical Reaction Behavior by Rational Design of a Red Phosphorus Electrode for Sodium-Ion Batteries
Source: Nanomaterials (Basel). 2021 Nov 13;11(11):3053. doi: 10.3390/nano11113053 (PMC8625586; doi:10.3390/nano11113053)
Supplement: Supplementary file 1 [file nanomaterials-11-03053-s001.zip › nanomaterials-1452969-supplementary.pdf]

nanomaterials

MDPI

Article

Rational Design for Electrochemical Analysis of a Red Phosphorus Electrode for Sodium-ion Batteries

Jong Hyuk Yun, San Moon, Do Kyung Kim* and Joo-Hyung Kim*

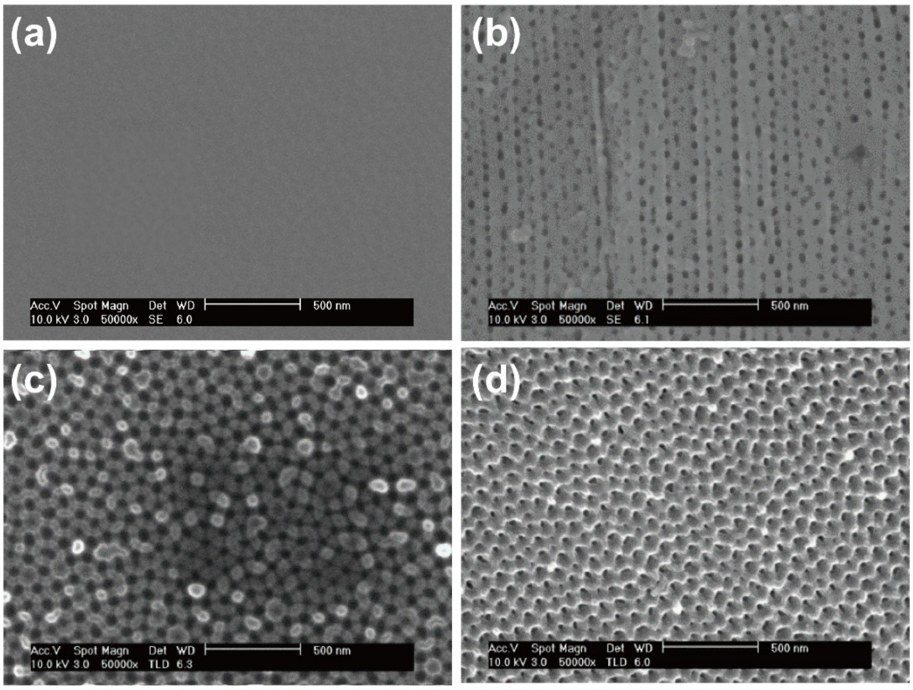

Figure S1. Procedure for fabrication of the red P@C NW electrode: (**a**) Electropolishing of the aluminum surface; (**b**) Preparation of porous alumina templates by the first-step anodization process; (**c**) Alumina etching process; (**d**) Preparation of porous alumina templates by a second-step anodization process.
